# Characterization of *Leishmania infantum* Isolates from Wild Leporids in the Community of Madrid (Spain)

**DOI:** 10.3390/vetsci13010001

**Published:** 2025-12-19

**Authors:** María Victoria Ortega-García, Nerea García, Mercedes Domínguez, Inmaculada Moreno

**Affiliations:** 1 Subdirección General de Sistemas Terrestres, Instituto Nacional de Técnica Aeroespacial (INTA)—Campus de La Marañosa, San Martín de la Vega, 28330 Madrid, Spain; ortegagmv@inta.es; 2 Centro de Vigilancia Sanitaria Veterinaria (VISAVET), Universidad Complutense de Madrid (UCM), 28040 Madrid, Spain; ngarciab@ucm.es; 3 Unidad de Inmunología Microbiana, Área de Inmunología, Instituto de Salud Carlos III (ISCIII), Majadahonda, 28220 Madrid, Spain; mdominguez@isciii.es

**Keywords:** molecular characterization, kDNA, PCR-RFLP, *Leishmania infantum*, wild animals, lagomorphs

## Abstract

In this research, 59 samples from 31 animals (19 European rabbits, 11 Iberian hares, and 1 cat) and an axenic culture of the *Leishmania infantum* isolate (MCAN/ES/97/10445, zymodeme ZM/MON-1) used as a reference were studied based on the analysis of kinetoplast minicircle (kDNA) restriction fragments by combining polymerase chain reaction and length polymorphisms (PCR-RFLP). This analysis was performed in parallel with polyacrylamide gel electrophoresis (PAGE) and capillary electrophoresis (CE), as well as in silico digestion of the abovementioned reference. These analyses did not reveal differences between the *L. infantum* isolates detected in the different samples of wild lagomorphs (rabbits and hares) from various areas of the Community of Madrid or with the axenically cultured promastigotes of the *L. infantum* isolate (MCAN/ES/97/10445, zymodeme ZM/MON-1) used as a reference. Consequently, it was proven that with the implemented approaches, only one isolate of *L. infantum* was responsible for infection in wild leporids and that these animals sustained the pathogen’s life cycle, both in the area of the human leishmaniasis outbreak that has been occurring in the Community of Madrid since 2009 and outside of it. Additionally, this isolate has been circulating since at least the 1990s.

## Simple Summary

Despite the public health implications of limited awareness regarding the biodiversity of *Leishmania* spp. and the persistence of human leishmaniasis outbreaks in endemic regions of Europe, there is still no consensus on a standardized molecular typing method for the surveillance of this parasite in humans, domestic animals, and wildlife reservoirs. Consequently, the development of a simple, rapid, effective, and cost-efficient typing approach remains an unmet need. The method proposed in this study, evaluated on a larger number of samples than in previous reports, may fulfill these criteria and contribute to improved molecular surveillance of *Leishmania* spp.

## 1. Introduction

Leishmaniasis is a neglected disease with a worldwide distribution and is endemic in countries of the Mediterranean basin. In developing countries, an estimated 350 million people are at risk of infection, the global prevalence is about 12–15 million cases, and between 700,000 and 1 million new cases are reported per year [1,2].

The main reservoir of *Leishmania infantum* is the domestic dog, although wild mammals such as rodents, mustelids, and canids also act as competent reservoirs of the parasite [3]. In the 2009 Community of Madrid outbreak of human leishmaniasis, considered the largest described in Europe up to that time, lagomorphs (hares and rabbits) were shown to be responsible for maintaining the circulation of the parasite [4,5,6,7,8,9,10,11,12,13]. Subsequently, the presence of *Leishmania* in lagomorphs has been reported in other autonomous communities [3,4,14,15,16] and in other countries of the Mediterranean basin, such as Italy [17] and Greece [18,19,20], highlighting the importance of this reservoir in the maintenance of the disease.

DNA of *L. infantum* has been detected by qPCR in samples of wild leporids (rabbits and hares) from several green areas in the capital of the region, outside the 2009 human leishmaniasis outbreak zones, as described in Ortega et al. [21]. The identification and comparison of strains isolated from lagomorphs in different areas would help to increase the knowledge about the wild cycle of this parasite [14].

The analysis of restriction fragments by combining polymerase chain reaction and length polymorphisms (PCR-RFLP) involves using specific restriction endonucleases to cleave the amplified genomic DNA of an isolate. The resulting fragments are then separated by electrophoresis according to their size. Mutations in the genome can alter the number of cut sites, while indels (insertions/deletions) can by changing their positions and lead to polymorphisms in fragment length. This method is simple and fast compared to others, such as isozyme analysis. Therefore, the kDNA PCR-RFLP combination is a useful technique for the molecular characterization of *Leishmania* spp. isolates [22,23,24,25].

In this study, a molecular characterization of *L. infantum* isolates from wild leporids (rabbits and hares) was carried out using PCR-RFLP to increase the knowledge about the circulation of this parasite in wildlife in Spain. In parallel, two methods of result analysis were compared: the traditional analysis using polyacrylamide gel electrophoresis (PAGE) and capillary electrophoresis (CE), with the latter providing better resolution.

## 2. Materials and Methods

### 2.1. Study Population and Sample Collection

**Figure 1:**
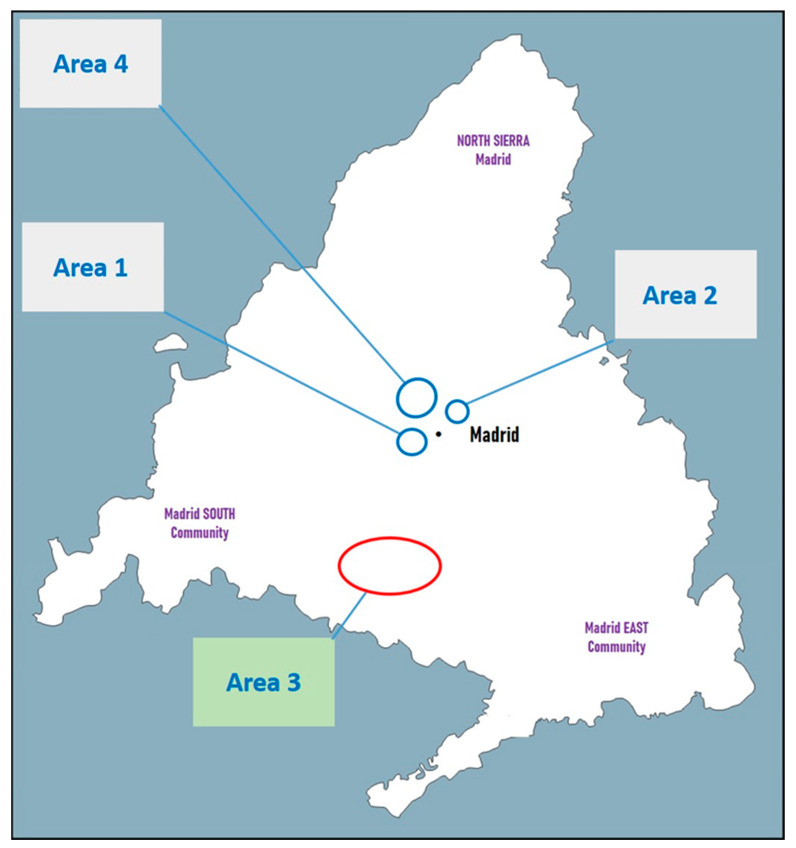
Schematic representation of the origin of the study population in the Community of Madrid (Spain). Outbreak area: area 3 (urbanized area with green spaces); non outbreak areas: areas 1 (green area), 2 (green area) and 4 (urbanized area with green spaces). The data were entered into OpenStreetMap.

Samples from the spleen, skin, and/or hair of 31 animals were analyzed: 19 European rabbits (*Oryctolagus cuniculus*), 11 Iberian hares (*Lepus granatensis*), and 1 cat (*Felis silvestris catus*). In addition, an axenic culture of *L. infantum* was included. The animals were collected from four geographically separated areas of the Community of Madrid. First, 15 rabbits and 5 hares were captured by the Madrid City Council authorities between September and November 2013 in two green areas outside of those affected by the 2009 human leishmaniasis outbreak. Fourteen rabbits were captured in the first area (area 1; green area), while 1 rabbit and 5 hares were captured in the second area (area 2; green area). Additionally, samples from 4 rabbits and 6 hares captured in 2014 in the outbreak area (area 3; urbanized area with green spaces) were analyzed in this study (Figure 1). As abovementioned, the isolate from a wild cat captured in 2013 in an area outside the outbreak (area 4; urbanized area with green spaces) and a reference strain of *L. infantum*, [MCAN/ES/97/10,445, ZM/MON-1 zymodeme], maintained in experimental hamster infections and in axenic cultures of 1, 9, and 103 passages (samples P1, P9, and P103, respectively), were included. From these animals (captured out of the original outbreak area: areas 1, 2, and 4; and captured in the original outbreak area: area 3), nucleic acid extraction from promastigotes obtained from culture of a portion of the spleen and/or from spleen tissue, skin, and/or hair was performed (Table 1).

Animals from the outbreak areas were sacrificed and frozen for storage until transfer to the Veterinary Health Surveillance Center (VISAVET), Complutense University of Madrid (UCM), Madrid (Spain), where the different samples were taken.

The sacrifice of the animals from areas not belonging to the outbreak, as well as the collection of all samples, regardless of their area of origin, was carried out in the same way as described in Ortega et al. [21]. The number of animals, the form of capture, and the areas studied were determined by the competent authority of the Madrid City Council in the context of a surveillance and control program. After capture, the animals were immediately transported to VISAVET, where they were euthanized and necropsied, and several samples (including blood, spleen, skin, and hair from the pinna) were collected. For in vitro culture, a piece of the spleen sample was immediately used; the blood was allowed to clot, and the skin, hair, and spleen samples that were not used for the culture were stored at −20 °C until processing by the chosen technique.

### 2.2. Isolation

A piece of the spleen sample was used for in vitro culture of nine animal samples and processed as described in Ortega et al. [21]. Concisely, the tissue was first homogenized in supplemented Schneider’s Drosophila medium, Sigma-Aldrich (10% heat-inactivated fetal bovine serum, HyClone, Thermo Fisher Scientific, Waltham, MA, USA; 50 mg/mL streptomycin and 100 U/mL penicillin, Lonza, Basel, Switzerland; 20 mM HEPES, Sigma-Aldrich, St. Louis, MO, USA; and 1% sterile urine), using a tissue grinder and then the homogenate was brought to 10 mg of tissue per ml of medium. The homogenate was cultured at 27 °C for a fortnight. Finally, promastigote growth was visualized using a fluorescence inverted microscope at 200× enlargement.

### 2.3. Molecular Analysis

#### 2.3.1. Extraction of Genomic Material, Real-Time PCR Amplification (qPCR), and Sanger Sequencing

Approximately 10 mg of spleen, 25 mg of skin, and 20–50 single hairs per animal were used for DNA extraction using the QIAamp Blood and Tissue Kit (QIAGEN, Hilden, Germany), following the manufacturer’s instructions. The extracted DNA was eluted in 150 µL and stored at −80 °C until use.

The TaqMan-MGB probe (FAM-5′-AAAAATGGGTGCAGAAAT-3′-nonfluorescent quencher-MGB) and the primers LEISH-1 (5′-AACTTTTCTGGTCCTCCGGGTAG-3′) and LEISH-2 (5′-ACCCCCAGTTTCCCGCC-3′) were used in PCR to amplify a 120-base-pair fragment of the conserved region in the *Leishmania* kinetoplast DNA minicircle (kDNA). Ten microliters of the QuantiTect^®^ Probe RT-PCR Master Mix Kit (QIAGEN, Hilden, Germany), 0.9 microliters of each primer (20 µM), 0.2 microliters of probe (20 µM), and 3 microliters of DNA were consumed in a total reaction volume of 20 µL for every reaction. The thermocycling protocol was as follows: 50 °C for 2 min, 95 °C for 10 min, and 40 cycles at 95 °C for 15 s and 60 °C for 1 min. A CFX Connect thermocycler (Bio-Rad, Hercules, CA, USA) was used for all reactions [21]. Additionally, Sanger sequencing was performed on 43 of the lagomorph samples (31 rabbits and 12 hares) and on the wild cat. Amplified products were enzymatically purified using the Illustra ExoProStar 1-STEP Kit (GE Healthcare Life Sciences, Chalfont St Giles, Buckinghamshire, UK). The BLASTn v2.7.1 tool was used to compare the obtained sequences with those available in the GenBank database. Sequencing was carried out at the Genomics Unit, ISCIII, Majadahonda, Madrid (Spain), following its own protocol.

#### 2.3.2. Standard PCR Amplification

The standard PCR procedure used was described previously [26] but was slightly modified (reduction of the annealing–elongation temperature from 62 °C to 56 °C). This PCR amplification was performed using the primers RV1 (5′-CTTTTCTGGTCCCGCGGGTAGG-3′) and RV2 (5′-CCACCTGGCCTATTTTACACCA-3′) to amplify a 145 bp fragment present in the *L. infantum* kDNA. In an iCycler thermal cycler (Bio-Rad, CA, USA), 12.5 µL (2×) of the Taq PCR Master Mix Kit (Qiagen, Hilden, Germany) and 0.5 µL of each primer (20 µM) were used in the amplification reaction. A final volume of 25 µL was obtained by adding 5 µL of template DNA. To visualize the PCR results, electrophoresis was carried out on a 2% agarose gel, including a DNA length standard. SYBR Safe (Invitrogen, Thermo Fisher Scientific, Waltham, MA, USA) was used as the nucleic acid stain, and the PCR 100 bp Low Ladder (Sigma-Aldrich, Merck KGaA, Darmstadt, Germany) was used as the molecular weight marker. Standard PCR was carried out at the Neglected and Emerging Diseases Unit (NED), VISAVET. The reagents used, volumes, and concentrations are shown in Table 2, while the conditions of the amplification reaction are described in Table 3.

#### 2.3.3. Restriction Fragment Length Polymorphism (RFLP)

Two restriction reactions were carried out on the PCR product corresponding to the 145 bp amplified fragment for each of the 59 samples analyzed, adapting the procedure previously described by Ferroglio et al. [27]. Twenty microliters of PCR products were digested separately with the restriction enzymes *Bsl*I (restriction site or target: 5′-CCNNNNN/NNGG-3′) and *Msc*I (restriction site or target: 5′-TGG/CCA-3′). Digestion was performed following the manufacturer’s instructions (NEB–New England Biolabs, Hitchin, Hertfordshire, UK) with slight modifications, using 1 μL of enzyme (*Bsl*I 10,000 units/mL and *Msc*I 5000 units/mL) in a 25 µL total reaction volume overnight.

#### Polyacrylamide Gel Electrophoresis (PAGE)

A total of 20 μL of the mixture, composed of 20 μL of digested product, 5 μL of 5x nucleic acid sample loading buffer (Bio-Rad), and 2 μL of SYBR Safe DNA Gel Stain (Invitrogen), was separated on a 15% Criterion TBE gel (18-well, 30 μL; Bio-Rad) via gel electrophoresis, and the fragment size was estimated by comparison with DNA standards: Molecular markers 20 bp Molecular Ruler (20–1000 bp; Bio-Rad) and AmpliSize (50–2000 bp; Bio-Rad) using SYBR Safe (Invitrogen). Polyacrylamide gel electrophoresis was carried out at the Microbial Immunology Unit, Instituto de Salud Carlos III (ISCIII), Majadahonda, Madrid (Spain).

#### Capillary Electrophoresis (CE)

Parallel to the PAGE technique, automated capillary electrophoresis (CE) was performed in the QIAxcel Advanced System (Qiagen), with the restriction fragments obtained from the 59 samples, in combination with the QIAxcel DNA High Resolution Kit (Qiagen), using the OM800 or OL800 method, depending on whether the estimated DNA concentration of the samples was above or below 10 ng/µL, respectively. The QX DNA 15 bp/600 bp standard was used as the alignment marker, and the QX 25 bp–500 bp standard (Qiagen) was used as the size marker. The data were analyzed using QIAxcel ScreenGel version 1.4.0 software. After processing the samples, the data were presented as simulated bands on a gel image and as peaks on electropherograms. Capillary electrophoresis was carried out in the Biological Defense Area, Department of CBRN Defense Systems, General Subdirectorate of Terrestrial Systems, INTA–Campus de La Marañosa, San Martín de la Vega, Madrid (Spain).

#### In Silico Digestion

In addition to the analysis of the restriction fragments using the PAGE and CE techniques, digestion was performed via computational simulation using DNASTAR Lasergene 13 version 11.1 software (DNASTAR, Madison, WI, USA) on a 140 bp sequence of the *L. infantum* reference strain MCAN/ES/98/10,445, which was deposited in the genetic sequence database of the National Institutes of Health (NIH) (USA) (GenBank accession number: EU437406.1).

## 3. Results

**Figure 2:**
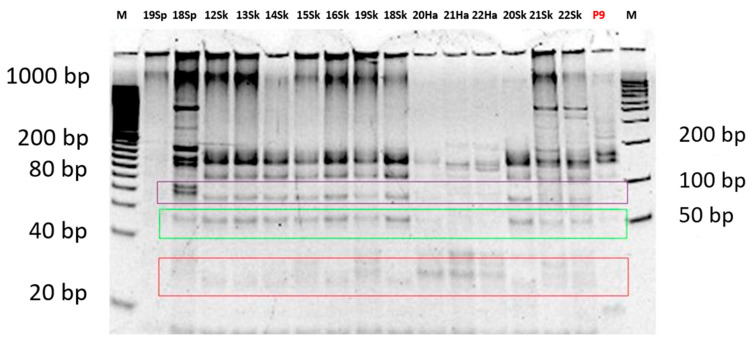
Image of the gel with the band patterns obtained for the restriction enzyme *Bsl*I after analysis by PCR-RFLP (PAGE). P9: pass 9 of the reference strain (in red). M: molecular weight marker. 22 or 28 bp bands (framed in red); 53 bp bands (framed in green); 70 bp bands (framed in purple).

**Figure 3:**
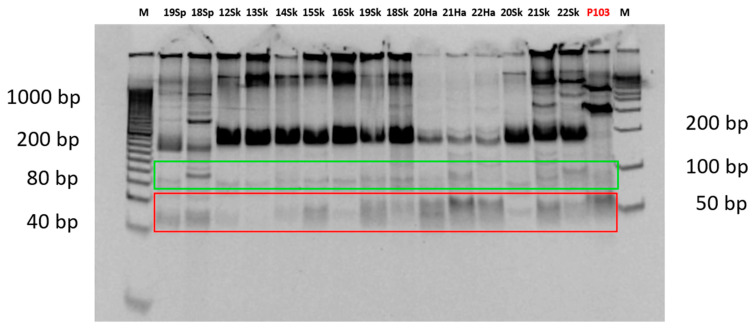
Image of the gel with the band patterns obtained for the restriction enzyme *Msc*I after analysis by PCR-RFLP (PAGE). P103: passage 103 of the reference strain (in red). M: molecular weight marker. 50 bp bands (framed in red); 66 and 79 bp bands (framed in green).

During the analysis of the restriction fragments using the PAGE technique, in the samples in which it was possible to determine (97%) because a clear band pattern was obtained, two major patterns for the restriction enzyme *Bsl*I were observed. The first pattern, with 3 bands of approximately 22 bp, 53 bp and 70 bp, was observed in 49% of the samples (29/59), whereas the second pattern, with 4 bands of approximately 22 bp, 28 bp, 53 bp and 70 bp, was detected in 44% of the samples (26/59) (Figure 2 and Table S1). For the restriction enzyme *Msc*I, a single majority 3-band pattern of approximately 50 bp, 66 bp and 79 bp was observed in 83% of the samples (49/59) (Figure 3 and Table S1). In the remaining samples, no clear pattern of bands could be detected for either of the two restriction enzymes. This majority pattern was designated as a “3–4/3” (3 or 4 bands for *Bsl*I, and 3 bands for *Msc*I). This pattern was maintained in both samples of axenic promastigotes of the reference strain used and in animal samples, regardless of the area from which they originated, the host, or the analyzed tissue. No differences were found in the band patterns between the passages by axenic culture of the promastigotes of the reference strain used as a positive control.

**Figure 4:**
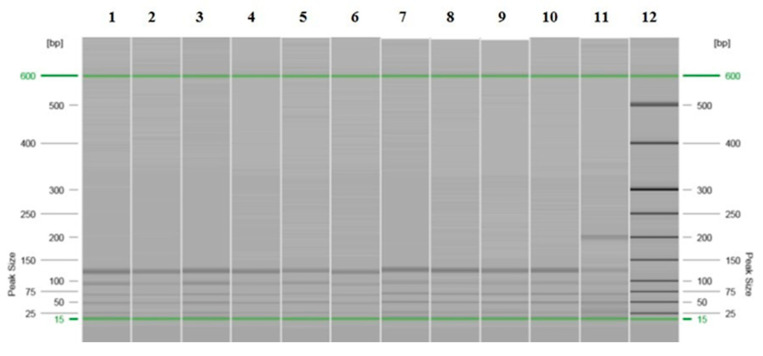
Image of the gel with the band patterns obtained for the restriction enzyme *Bsl*I after analysis by PCR-RFLP (CE). Promastigotes were isolated from the spleen (lanes 1–9), passage 9 (lane 10), and passage 103 (lane 11) of the reference strain and from the molecular weight marker (lane 12). The green lines correspond to the QX DNA 15 bp/600 bp alignment markers.

**Figure 5:**
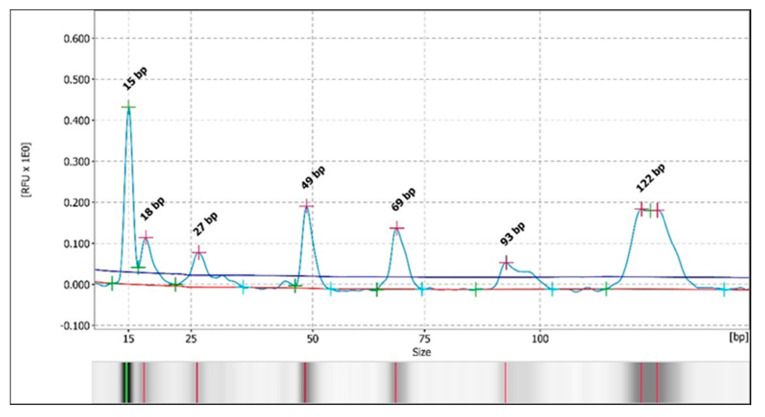
Electropherogram with the peak patterns obtained for the restriction enzyme *Bsl*I after analysis by PCR-RFLP (CE). Sample 9Is. The red lines correspond to the fragment sizes expressed in base pairs (bp).

**Figure 6:**
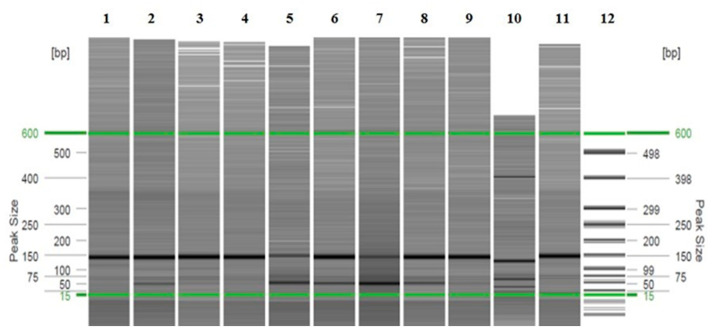
Image of the gel with the band patterns obtained for the restriction enzyme *Msc*I after analysis by PCR-RFLP (CE). Spleen isolates (lanes 1, 4, 5, 6 and 11), spleen (lanes 2 and 7), skin (lanes 3, 8 and 10), and passage 9 (lane 9) of the reference strain and molecular weight marker (lane 12). The green lines correspond to the QX DNA 15 bp/600 bp alignment markers.

**Figure 7:**
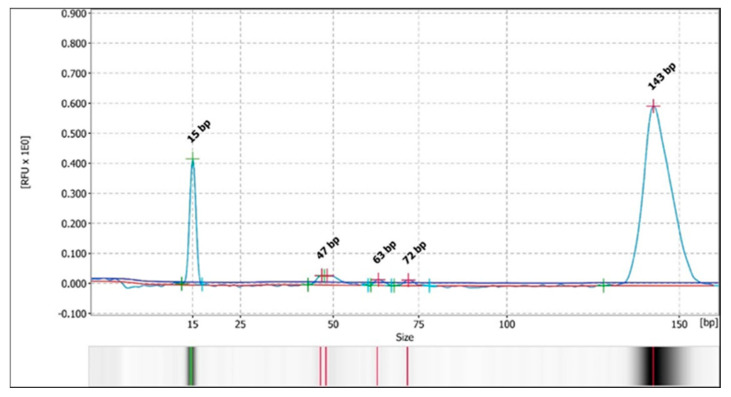
Electropherogram with the peak patterns obtained for the restriction enzyme *Msc*I after analysis by PCR-RFLP (CE). Sample 3Sk. The red lines correspond to the fragment sizes expressed in base pairs (bp).

**Figure 8:**
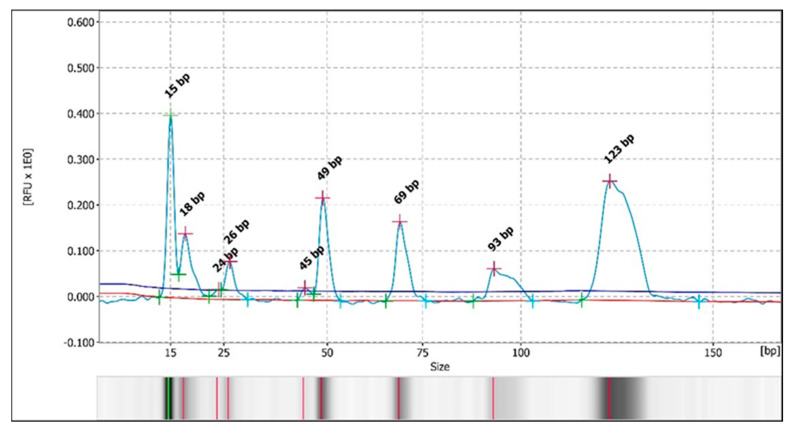
Electropherogram with the peak patterns obtained for the restriction enzyme *Bsl*I after analysis by PCR-RFLP (CE). Sample P9. The red lines correspond to the fragment sizes expressed in base pairs (bp).

**Figure 9:**
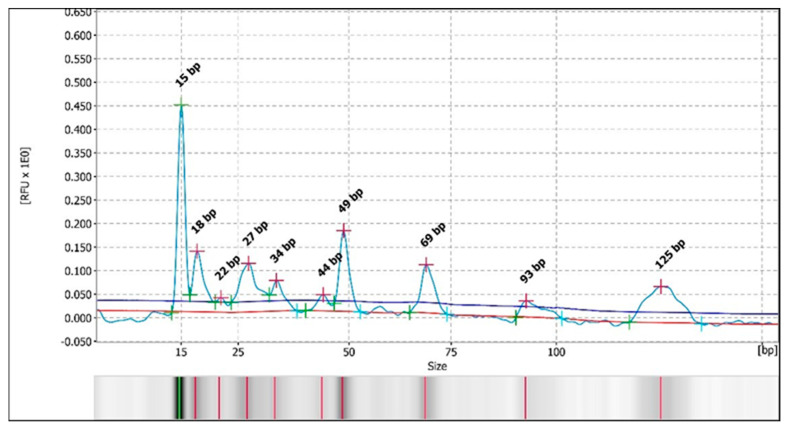
Electropherogram with the peak patterns obtained for the restriction enzyme *Bsl*I after analysis by PCR-RFLP (CE). Sample P103. The red lines correspond to the fragment sizes expressed in base pairs (bp).

Otherwise, during the analysis of the restriction fragments by automated CE, in the samples in which it could be determined (68%), the same pattern of bands/peaks was observed as that detected with the PAGE technique. A pattern of 3–4 bands for the *Bsl*I enzyme was detected in 58% of the samples (34/59) (Figure 4 and Figure 5 and Table S1), with two major patterns, and a 3-band pattern for the *Msc*I enzyme was detected in 29% of the samples (17/59 samples) (Figure 6 and Figure 7 and Table S1) in all of the previously stated conditions (by species, by study area or origin, and by sample within the same animal), with a difference of ± 3–5 bp with respect to the predicted sizes according to in silico digestion and/or the PAGE technique (see Table S1). Furthermore, CE allowed us to observe more clear differences in the pattern of bands obtained with the *Bsl*I enzyme between the promastigotes of the reference strain that had 9 or 103 passages per axenic culture; therefore, successive subcultures of *Leishmania* may alter their PCR-RFLP profile (Figure 8 and Figure 9 and Table S1). CE also confirmed the pattern of bands obtained by the PAGE technique with the *Bsl*I enzyme in 33 out of 59 samples and in 16 out of 59 samples with the *Msc*I enzyme, and it was additionally able to discern the pattern of bands in two samples—one with the *Bsl*I enzyme and another with *Msc*I—in which it could not be determined using the PAGE technique.

**Figure 10:**
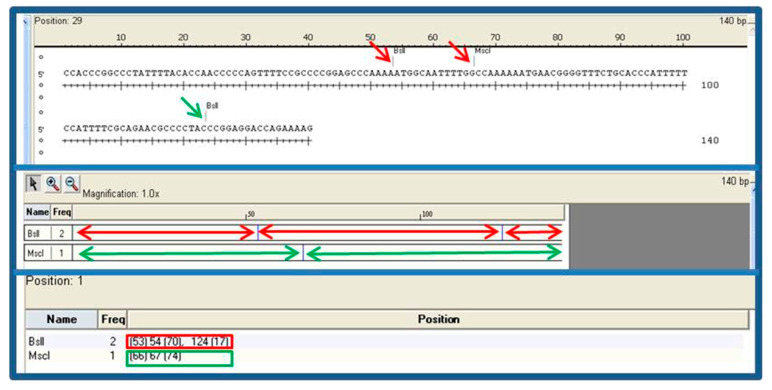
In silico digestion of the fragment of the *L. infantum* strain MCAN/ES/98/10,445 (GenBank accession number: EU437406.1) by means of the restriction enzymes *Bsl*I and *Msc*I.

When we analyzed the theoretical band profile by means of in silico digestion (Figure 10), we observed differences from the profile obtained by PAGE and by CE with the two restriction enzymes employed, both in the samples of wild lagomorphs and in the strain that was used as the reference (*L. infantum* MCAN/ES/97/10,445). In silico digestion with the *Bsl*I enzyme gave rise to three bands of 17, 53, and 70 bp, and with the *Msc*I enzyme, two bands of 66 and 74 bp were observed instead of those obtained via PAGE and CE analysis.

Additionally, qPCR followed by Sanger sequencing was performed on 43 of the lagomorph samples (31 rabbits and 12 hares) and on the wild cat (Table S2). These sequences showed homology with the *L. infantum* MCAN/ES/98/10,445 isolates deposited at the National Institutes of Health (NIH) (clone LinGpja_8, complete kinetoplast minicircle sequence; GenBank accession numbers EU437406.0, EU437406.1, EU437406.2, and EU437406.3) ranging from 78% to 98%, with greater than or equal to 96% identity in 44 of the sequenced samples (66%) (Table S1).

During the analysis of the restriction fragments, bands/peaks other than those predicted were observed both with the PAGE technique and with CE.

## 4. Discussion

Comparing both types of RFLP analysis, automated CE allowed for the analysis of fragments digested by restriction enzymes in a more reproducible way and the estimation of DNA fragment sizes more rapidly than with the PAGE technique [28,29]. Moreover, with the PAGE technique, the presence of weak bands/peaks was also observed in some of the samples analyzed, which made their interpretation difficult. This could be the consequence of a low DNA concentration in the sample, as previously reported by other authors [30]. CE even allowed us to observe differences in promastigotes that were kept in axenic culture for more than 100 passages. The alterations that *Leishmania* undergoes when maintained in culture, and their implications for the sensitivity of serological analyses, had already been observed in previous work by our group [6], but now we have a fast and reliable method that allows us to analyze the quality of the promastigotes used as antigenic targets.

The similarities found between the profile observed with both restriction enzymes in the samples of wild lagomorphs and those observed in the reference strain not only confirm that they correspond to the same strain but also indicate that the strain involved in the outbreak, as well as the one maintained in parasite’s wild cycle by the lagomorphs in the Community of Madrid, may have been circulating for at least two decades, considering the reference strain code *L. infantum* MCAN/ES/97/10,445.

Regarding the patterns observed, authors such as [16] obtained 4 different band patterns by PCR-RFLP, also following the procedure described by Ferroglio et al. [27], in isolates of *L. infantum* from free-rearing hares from the Spanish geographic region called “Centro” in that study (which would include part of the Community of Madrid), which were found dead or killed by hunters between 2004 and 2010. However, other researchers found a single pattern, as in this study—a type called ITS-LOMBARDI—by sequencing the ITS1 and ITS2 regions of *L. infantum* isolates obtained by xenodiagnosis from hares related to the outbreak of human leishmaniasis in the Community of Madrid [4]. In other similar studies on this outbreak in Spain, but published more recently, the authors reached a similar conclusion despite using typing methods different from those employed in the present study and in the one abovementioned [31,32]. This type is also the only one observed in the isolates from all human cases associated with this outbreak that have been typified to date [7]. According to Chicharro et al., the ITS-LOMBARDI genotype has been frequently found in *L. infantum* isolates from cases of human leishmaniosis in the Community of Madrid, where it is considered to have been circulating since the 1990s.

More recently, it has been reported that although the kDNA PCR-RFLP used in their study was less able to discriminate among *L. infantum* strains than SNP genotyping, this technique could group different *L. infantum* strains comparably [15]. Additionally, their results showed that there was a genotype (genotype B) present simultaneously in humans, dogs, and wildlife. Both findings support, on the one hand, the use of kDNA PCR-RFLP in the characterization of *L. infantum* strains and, on the other hand, the possibility of the strain being part of the same transmission cycle.

There are two possible explanations for the presence of additional bands/peaks. The first is that it may be due to incomplete digestion by the restriction enzyme; among the most likely causes in this study are: (i) a slower rate of fragment breakage than expected, which could be solved by increasing the enzyme incubation time, and (ii) DNA contamination with an inhibitor, which could be solved by passing the DNA through a purification column. The second explanation may be the presence of extra bands in the gel; among the most likely causes in this study are the following: (i) the enzyme may have bound to the substrate used during electrophoresis, which could be solved by reducing the number of enzyme units during digestion; (ii) the presence of star activity (only in the case of the *Msc*I enzyme, as it is the only one of the two enzymes with this activity), which could be solved by reducing the number of enzyme units in the reaction—ensuring that the amount of enzyme does not exceed 10% of the total reaction volume— and by reducing the incubation time; and (iii) partial digestion by the restriction enzyme, which could be solved in the same way as incomplete digestion. The procedure followed for digestion with the restriction enzymes was a compromise between the characteristics of the samples and the manufacturer’s recommendations, so it was not possible to address all causes without sacrificing solutions to others. Among the possible solutions recommended by the manufacturer, the following were applied: DNA extraction was carried out using the columns provided with the extraction kit, the volume of enzyme used did not exceed 10% of that recommended by the manufacturer, and digestion was carried out overnight. A previous optimization was carried out via the PAGE technique with the *Bsl*I enzyme, using increasing amounts of PCR product and increasing amounts of enzyme, and the best results were obtained with 20 µL of product and 1 µL of enzyme.

The differences observed by in silico digestion could be due to the fragment analyzed in the strain deposited in GenBank being shorter (140 bp) than the one amplified during molecular characterization (145 bp) and to the fact that, with fewer base pairs, another recognition sequence for the enzyme may appear, resulting in an additional cut of the fragment.

Regarding the limitations of this study—such as factors influencing the isolate’s long-term circulation (e.g., vector dynamics, environmental drivers, etc.)—additional studies with a larger number of samples are needed.

## 5. Conclusions

No differences were detected by minicircle kDNA PCR-RFLP analysis between promastigotes isolated from wild leporid tissues collected in various green areas of the Community of Madrid and promastigotes from the reference *L. infantum* strain (MCAN/ES/97/10,445, ZM/MON-1 zymodeme). Therefore, a single isolate circulating since at least the 1990s may be responsible for infection in lagomorphs, and these animals support the wild cycle of the disease.

The kDNA PCR-RFLP results and the sequencing data supporting this study’s findings are provided in the Supplementary Materials.

The RFLP technique has been confirmed as a method that allows for the characterization of different *Leishmania* isolates, especially when the analysis is performed using capillary electrophoresis.

## Figures and Tables

**Table 1:** List of animals and samples analyzed by origin and their relationship with the 2009 outbreak. Promastigotes were isolated from spleen (Is), spleen tissue (Sp), skin (Sk), and hair (Ha) samples.

Identification Code (ID)	Species (Samples)	Origin (Area)	Related with the Outbreak
1	Reference strain (Is)	Instituto de Salud Carlos III	No
2	Cat (Is)	4	No
3	Rabbit (Is, Sp, Sk)	1	No
4	Rabbit (Is, Sp, Sk)	1	No
5	Rabbit (Is, Sp, Sk)	1	No
6	Rabbit (Is, Sp, Sk)	1	No
7	Rabbit (Is, Sp, Sk)	1	No
8	Rabbit (Is, Sp, Sk)	1	No
9	Rabbit (Is, Sp)	1	No
10	Rabbit (Is, Sp)	1	No
11	Rabbit (Is)	1	No
12	Rabbit (Sp, Sk)	1	No
13	Rabbit (Sp, Sk)	1	No
14	Rabbit (Sp, Sk)	1	No
15	Rabbit (Sp, Sk)	1	No
16	Rabbit (Sp, Sk)	1	No
17	Rabbit (Sk)	2	No
18	Hare (Sp, Sk)	2	No
19	Hare (Sp, Sk)	2	No
20	Hare (Sk, Ha)	2	No
21	Hare (Sk, Ha)	2	No
22	Hare (Sk, Ha)	2	No
23	Hare (Sk)	3	Yes
24	Hare (Sk)	3	Yes
25	Hare (Sk)	3	Yes
26	Hare (Sk)	3	Yes
27	Hare (Sk)	3	Yes
28	Hare (Sk)	3	Yes
29	Hare (Sk)	3	Yes
30	Hare (Sk)	3	Yes
31	Hare (Sk, Ha)	3	Yes
32	Hare (Sk)	3	Yes

**Table 2:** Composition, volumes and concentrations of the PCR mixture.

Reagent	Initial Concentration	Volume per Sample	Final Concentration
PCR grade water		6.5 µL	
Primer RV1	20 µM	0.5 µL	0.4 µM
Primer RV2	20 µM	0.5 µL	0.4 µM
Taq PCR Master Mix	2×	12.5 µL	1×

**Table 3:** Amplification conditions.

Number of Cycles	PCR Steps	Temperature	Time
1 cycle	Initial denaturation	95 °C	15 min
45 cycles	Denaturation	94 °C	1 min
	Hybridization	56 °C	1.5 min
	Extension	70 °C	45 s (with an increase of 5 s each cycle)
1 cycle	Final extension	72 °C	10 min

## Data Availability

The original contributions presented in this study are included in the article/Supplementary Materials. Further inquiries can be directed to the corresponding author.

## Supplementary Materials

The following supporting information can be downloaded at: https://www.mdpi.com/article/10.3390/vetsci13010001/s1, Table S1: Summary of the results of the analysis by PCR-RFLP (PAGE and CE) and sequencing by species and origin of the samples; Table S2: Summary of the results of the analysis by qPCR.
