# Supplementary material for: Characterization of Leishmania infantum Isolates from Wild Leporids in the Community of Madrid (Spain)
Source: Vet Sci. 2025 Dec 19;13(1):1. doi: 10.3390/vetsci13010001 (PMC12846632; doi:10.3390/vetsci13010001)
Supplement: Supplementary file 1 [file vetsci-13-00001-s001.zip › vetsci-3979364-supplementary.pdf]

Table S1. Summary of the results of the analysis by kDNA PCR-RFLP (PAGE and CE) and sequencing, by species and origin of the samples.

ID	PAGE		EC		Sample	Origin	Sequencing result	*GenBank number*	Homology (%))
	Fragments		Fragments						
	*Bsl*I	*Msc*I	*Bsl*I	*Msc*I					
1	(22, 53, 70) 3		(18, 28, 53, 74) 4		Reference strain* (Is), P1	ISCIII			
	(22, 28, 53, 70) 4	(50, 66, 79) 3	(18, 24, 49, 69) 4	(45, 62, 71) 3	Reference strain* (Is), P9	ISCIII			
		(50, 66, 79) 3	(18, 27, 34, 49, 69) 5	(46, 67, 71) 3	Reference strain* (Is), P103	ISCIII	*L. infantum isolate MCAN/ES/98/10,445 clone LinGpja_8 kinetoplast minicircle, complete sequence*	EU437406.1	98 %
2	(22, 53, 70) 3	(50, 66, 79) 3			Cat (Is)	4	*L. infantum isolate MCAN/ES/98/10,445 clone LinGpja_8 kinetoplast minicircle, complete sequence*	EU437406.2	83 %
3	(22, 53, 70) 3	(66, 79) 2	(18, 26, 48, 68) 4	(62, 70) 2	Rabbit (Is)	1	*L. infantum isolate MCAN/ES/98/10,445 clone LinGpja_8 kinetoplast minicircle, complete sequence*	EU437406.1	79 %
	(22, 28, 53, 70) 4	(50, 66, 79) 3		(48, 62, 70) 3	Rabbit (Sp)	1			
	(22, 53, 70) 3	(66, 79) 2	(18) 1	(64, 72) 2	Rabbit (Sk)	1	*L. infantum isolate MCAN/ES/98/10,445 clone LinGpja_8 kinetoplast minicircle, complete sequence*	EU437406.1	98 %
4	(22, 53, 70) 3	(66, 79) 2	(18, 26, 48, 68) 4	(63, 72) 2	Rabbit (Is)	1	*L. infantum isolate MCAN/ES/98/10,445 clone LinGpja_8 kinetoplast minicircle, complete sequence*	EU437406.1	98 %
	(22, 28, 53, 70) 4	(50, 66, 79) 3	(18) 1	(51) 1	Rabbit (Sp)	1			
	(22, 53, 70) 3	(50, 66, 79) 3	(17) 1	(65, 75) 2	Rabbit (Sk)	1	*L. infantum isolate MCAN/ES/98/10,445 clone LinGpja_8 kinetoplast minicircle, complete sequence*	EU437406.1	91 %
5	(22, 53, 70) 3	(66, 79) 2	(18, 26, 49, 69) 4	(67, 76) 2	Rabbit (Is)	1	*L. infantum isolate MCAN/ES/98/10,445 clone LinGpja_8 kinetoplast minicircle, complete sequence*	EU437406.1	98 %
	(22, 28, 53, 70) 4	(50, 66, 79) 3	(18) 1	(47, 63, 72) 3	Rabbit (Sp)	1	*L. infantum*		84 %
	(22, 53, 70) 3	(50, 66, 79) 3	(18, 26, 48, 68) 4		Rabbit (Sk)	1			

6	(22, 53, 70) 3	(50, 66, 79) 3	(18, 26, 48, 68) 4	(49, 76) 2	Rabbit (Is)	1	*L. infantum isolate MCAN/ES/98/10,445 clone LinGpja_8 kinetoplast minicircle, complete sequence*	EU437406.1	98 %
	(22, 53, 70) 3	(50, 66, 79) 3		(51, 67, 74) 3	Rabbit (Sp)	1			
	(22, 53, 70) 3	(50, 66, 79) 3	(18) 1		Rabbit (Sk)	1	*L. infantum isolate MCAN/ES/98/10,445 clone LinGpja_8 kinetoplast minicircle, complete sequence*	EU437406.0	90 %
7	(22, 53, 70) 3	(66, 79) 2	(18, 27, 50, 70) 4	(76) 1	Rabbit (Is)	1	*L. infantum isolate MCAN/ES/98/10,445 clone LinGpja_8 kinetoplast minicircle, complete sequence*	EU437406.1	91 %
	(22, 28, 53, 70) 4	(50, 66, 79) 3	(18, 26, 49, 69) 4		Rabbit (Sp)	1	*L. infantum isolate MCAN/ES/98/10,445 clone LinGpja_8 kinetoplast minicircle, complete sequence*	EU437406.1	98 %
	(22, 53, 70) 3	(50, 66, 79) 3	(18, 26, 48, 68) 4	(45, 63, 72) 3	Rabbit (Sk)	1	*L. infantum isolate MCAN/ES/98/10,445 clone LinGpja_8 kinetoplast minicircle, complete sequence*	EU437406.1	98 %
8	(22, 28, 53, 70) 4	(66, 79) 2	(18, 25, 47, 67) 4	(63, 72) 2	Rabbit (Is)	1	*L. infantum isolate MCAN/ES/98/10,445 clone LinGpja_8 kinetoplast minicircle, complete sequence*	EU437406.1	98 %
	(22, 53, 70) 3	(50, 66, 79) 3	(18, 30, 51) 3	(56) 1	Rabbit (Sp)	1	*L. infantum isolate MCAN/ES/98/10,445 clone LinGpja_8 kinetoplast minicircle, complete sequence*	EU437406.1	98 %
	(22, 53, 70) 3	(66, 79) 2	(17, 26, 48, 67) 4		Rabbit (Sk)	1	*L. infantum isolate MCAN/ES/98/10,445 clone LinGpja_8 kinetoplast minicircle, complete sequence*	EU437406.1	98 %
9	(22, 28, 53, 70) 4	(50, 66, 79) 3	(18, 27, 50, 70) 4	(44, 62, 71) 3	Rabbit (Is)	1	*L. infantum isolate MCAN/ES/98/10,445 clone LinGpja_8 kinetoplast minicircle, complete sequence*	EU437406.1	98 %
	(22, 53, 70) 3	(50, 66, 79) 3	(18, 27, 48, 67) 4	(40, 60) 2	Rabbit (Sp)	1	*L. infantum isolate MCAN/ES/98/10,445 clone LinGpja_8 kinetoplast minicircle, complete sequence*	EU437406.2	79 %
10	(22, 28, 53, 70) 4	(50, 66, 79) 3	(18, 27, 49, 69) 4	(46, 63, 72) 3	Rabbit (Is)	1	*L. infantum isolate MCAN/ES/98/10,445 clone LinGpja_8 kinetoplast minicircle, complete sequence*	EU437406.1	98 %
	(22, 53, 70) 3	(50, 66, 79) 3	(18) 1		Rabbit (Sp)	1			
11	(22, 28, 53, 70) 4	(50, 66, 79) 3	(18, 27, 49, 69) 4	(49, 64, 73) 3	Rabbit (Is)	1	*L. infantum* isolate MCAN/ES/98/10445 clone LinGpja_8 kinetoplast minicircle, complete sequence	EU437406.1	98%
12	(22, 28, 53, 70) 4	(50, 66, 79) 3	(17, 27, 47) 3	(49, 63, 72) 3	Rabbit (Sp)	1	*L. infantum* isolate MCAN/ES/98/10445 clone LinGpja_8 kinetoplast minicircle, complete sequence	EU437406.2	88%

	(22, 53, 70) 3	(50, 66, 79) 3	(18, 27, 48) 3	(52, 71, 73) 3	Rabbit (Sk)	1	*L. infantum* isolate MCAN/ES/98/10445 clone LinGpja_8 kinetoplast minicircle, complete sequence	EU437406.0	89%
13	(22, 28, 53, 70) 4	(50, 66, 79) 3	(18, 27, 48) 3	(53) 1	Rabbit (Sp)	1	*L. infantum* isolate MCAN/ES/98/10445 clone LinGpja_8 kinetoplast minicircle, complete sequence	EU437406.1	98%
	(22, 53, 70) 3	(50, 66, 79) 3	(18, 26, 49, 68) 4	(50) 1	Rabbit (Sk)	1	*L. infantum isolate MCAN/ES/98/10,445 clone LinGpja_8 kinetoplast minicircle, complete sequence*	EU437406.2	80 %
14	(22, 28, 53, 70) 4	(50, 66, 79) 3	(18, 26, 48, 67) 4	(49) 1	Rabbit (Sp)	1	*L. infantum isolate MCAN/ES/98/10,445 clone LinGpja_8 kinetoplast minicircle, complete sequence*	EU437406.1	98 %
	(22, 53, 70) 3	(50, 66, 79) 3	(17, 27, 48) 3	(47) 1	Rabbit (Sk)	1	*L. infantum isolate MCAN/ES/98/10,445 clone LinGpja_8 kinetoplast minicircle, complete sequence*	EU437406.1	98 %
15	(22, 28, 53, 70) 4	(50, 66, 79) 3	(18, 27, 48, 68) 4	(49) 1	Rabbit (Sp)	1	*L. infantum isolate MCAN/ES/98/10,445 clone LinGpja_8 kinetoplast minicircle, complete sequence*	EU437406.2	84 %
	(22, 28, 53, 70) 4	(50, 66, 79) 3	(18) 1	(44, 62, 72) 3	Rabbit (Sk)	1	*L. infantum isolate MCAN/ES/98/10,445 clone LinGpja_8 kinetoplast minicircle, complete sequence*	EU437406.1	98 %
16	(22, 28, 53, 70) 4	(50, 66, 79) 3	(18) 1	(46, 58) 2	Rabbit (Sp)	1	*L. infantum isolate MCAN/ES/98/10,445 clone LinGpja_8 kinetoplast minicircle, complete sequence*	EU437406.2	(22, 28, 53, 70) 4
	(22, 53, 70) 3	(50, 66, 79) 3	(18, 26, 47) 3	(50, 64, 73) 3	Rabbit (Sk)	1			(22, 53, 70) 3
17			(17, 25, 47) 3	(49) 1	Rabbit (Sk)	2			
18	(22, 28, 53, 70) 4	(50, 66, 79) 3	(17) 1	(58, 74) 2	Hare (Sp)	2			
	(22, 53, 70) 3	(50, 66, 79) 3	(17, 25, 46, 64) 4	(60) 1	Hare (Sk)	2	*L. infantum isolate MCAN/ES/98/10,445 clone LinGpja_8 kinetoplast minicircle, complete sequence*	EU437406.1	98 %
19		(50, 66, 79) 3	(19) 1	(44, 65, 79) 3	Hare (Sp)	2	*L. infantum*		86 %
	(22, 28, 53, 70) 4	(50, 66, 79) 3			Hare (Sk)	2			
20	(22, 28, 53, 70) 4	(50, 66, 79) 3	(17, 24, 44, 62) 4	(55) 1	Hare (Sk)	2	*L. infantum isolate MCAN/ES/98/10,445 clone LinGpja_8 kinetoplast minicircle, complete sequence*	EU437406.1	98 %
	(22, 28, 53, 70) 4	(50, 66, 79) 3		(54, 68) 2	Hare (Ha)	2	*L. infantum isolate MCAN/ES/98/10,445 clone LinGpja_8*	EU437406.2	88 %

							kinetoplast minicircle, complete sequence		
21	(22, 28, 53, 70) 4	(50, 66, 79) 3	(18, 27, 48) 3		Hare (Sk)	2	*L. infantum isolate MCAN/ES/98/10,445 clone LinGpja_8 kinetoplast minicircle, complete sequence*	EU437406.3	79 %
	(22, 28, 53, 70) 4	(50, 66, 79) 3	(17) 1		Hare (Ha)	2			
22	(22, 28, 53, 70) 4	(50, 66, 79) 3	(18, 25, 48) 3	(47) 1	Hare (Sk)	2	*L. infantum isolate MCAN/ES/98/10,445 clone LinGpja_8 kinetoplast minicircle, complete sequence*	EU437406.1	98 %
	(22, 28, 53, 70) 4	(50, 66, 79) 3	(17) 1		Hare (Ha)	2			
23	(22, 53, 70) 3	(50, 66, 79) 3	(18) 1		Rabbit (Sk)	3	*L. infantum isolate MCAN/ES/98/10,445 clone LinGpja_8 kinetoplast minicircle, complete sequence*	EU437406.1	98 %
24	(22, 53, 70) 3	(50, 66, 79) 3	(18) 1	(48, 59) 2	Rabbit (Sk)	3	*L. infantum isolate MCAN/ES/98/10,445 clone LinGpja_8 kinetoplast minicircle, complete sequence*	EU437406.1	98 %
25	(22, 53, 70) 3	(50, 66, 79) 3	(17, 27, 47) 3	(49) 1	Rabbit (Sk)	3	*L. infantum isolate MCAN/ES/98/10,445 clone LinGpja_8 kinetoplast minicircle, complete sequence*	EU437406.1	98 %
26	(22, 53, 70) 3	(50, 66, 79) 3		(69, 77) 2	Rabbit (Sk)	3	*L. infantum isolate MCAN/ES/98/10,445 clone LinGpja_8 kinetoplast minicircle, complete sequence*	EU437406.2	78 %
27	(22, 53, 70) 3	(50, 66, 79) 3	(18, 25, 48, 68) 4	(56, 79) 2	Hare (Sk)	3	*L. infantum isolate MCAN/ES/98/10,445 clone LinGpja_8 kinetoplast minicircle, complete sequence*	EU437406.1	98 %
28	(22, 53, 70) 3	(50, 66, 79) 3	(17) 1	(47, 76) 2	Hare (Sk)	3	*L. infantum isolate MCAN/ES/98/10,445 clone LinGpja_8 kinetoplast minicircle, complete sequence*	EU437406.1	98 %
29	(22, 28, 53, 70) 4	(50, 66, 79) 3		(57, 67, 72) 3	Hare (Sk)	3	*L. infantum isolate MCAN/ES/98/10,445 clone LinGpja_8 kinetoplast minicircle, complete sequence*	EU437406.1	98 %

Promastigotes isolated from spleen (Is); spleen tissue (Sp), skin (Sk) and hair (Ha) samples; Reference strain passages 1 (P1), 9 (P9) and 103 (P103).

* Maintained in experimental infections in hamsters.

Table S2. Summary of the results of the analysis by qPCR.

ID	qPCR Result
1	Reference strain used as positive control in Ortega et al. 2017 [21]
2	Sp positive (data not found; only yes/no result)
3	Is (11.35), Sp (23.62), Sk (20.22)
4	Is (12.35), Sp (31.3), Sk (29.28)
5	Is (2.3), Sp (28.21), Sk (18.66)
6	Is (23.81), Sp (38.59), Sk (25.3)
7	Is (11.82), Sp (27.58), Sk (22.18)
8	Is (14.93), Sp (28.28), Sk (23.48)
9	Is (23.97), Sp (32.71), Sk (39.34)
10	Is (26.19), Sp (35.92), Sk (25.44)
11	Is (27.06), Sp (32.82), Sk (22.36)
12	Sp (33.76), Sk (28.06)
13	Sp (30.43), Sk(23.54)
14	Sp (32.05), Sk (29.03)
15	Sp (28.27), Sk (30.29)
16	Sp (33.34), Sk (23.77)
17	Sp (30.43), Sk (23.54)
18	Sp (30.43), Sk (23.54)
19	Sp (30.43), Sk (23.54)
20	Sk (25.73)
21	Sp (35.2), Sk (31.4), Ha (34.19)
22	Sk (31.2), Ha (35.73)
23	Sk (21.78), Ha (25.02)
24	Sk (21.44), Ha (32.05)
25	Sk (28.04), Ha (38.35)
26	Sk (29.06), Ha (38.03)
27	Sp (36.05), Sk (24.97), Ha (36,16)
28	Sp (29.77), Sk (15.57), Ha (25.15)
29	Sp (34.44), Sk (24.37), Ha (35.02)
30	Sp (34.76), Sk (26.1), Ha (38.93)
31	Sp (25.29), Sk (20.27), Ha (26.95)
32	Sp (28.7), Sk (20.71), Ha (21.82)

Promastigotes isolated from spleen (Is); spleen tissue (Sp), skin (Sk) and hair (Ha)
